# A novel frameshift mutation in *FRMD7* causes X-linked infantile nystagmus in a Chinese family

**DOI:** 10.1186/s12881-018-0720-8

**Published:** 2019-01-07

**Authors:** Junjue Chen, Yan Wei, Linlu Tian, Xiaoli Kang

**Affiliations:** Department of Ophthalmology in XinHua hospital, Shanghai Jiao Tong University, Kongjiang road 1665, Yangpu District, Shanghai, China

**Keywords:** Infantile nystagmus, Mutation, FERM domain-containing 7 (*FRMD7*) gene

## Abstract

**Background:**

Infantile nystagmus (IN) is an oculomotor disorder that is characterized by conjugate involuntary, rapid and repetitive movement of the eyes. To date, the pathogenesis of IN remains unclear. Many patients show an X-linked inheritance pattern. In this study, we explored the mutation in the FERM domain-containing 7 (*FRMD7*) gene in a Chinese family with X-linked infantile nystagmus.

**Methods:**

We conducted comprehensive ocular examinations and collected 5 ml of blood samples from members of a family with X-linked IN and 100 normal controls. Mutations in FRMD7 were identified by sequencing PCR products.

**Results:**

We found a 7-bp deletion(c.823-829delACCCTAC) in the 9th exon of FRMD7 in a Chinese family with IN, which predicted a truncation of the protein.

**Conclusions:**

This study reported a novel mutation of the FRMD7 gene occurred in a Chinese family with IN, thus expanding the spectrum of FRMD7 mutations causing IN, and further confirming that the mutations of FRMD7 are the underlying molecular cause of IN.

## Background

Infantile nystagmus(IN) is an oculomotor disorder that is characterized by conjugate involuntary, rapid and repetitive movement of the eyes. IN may be clinically apparent at birth or is noticed by parents within the first six months of patient’s life [1]. IN can appear along with ocular pathologies such as congenital cataracts, optic nerve hypoplasia or albinism. IN can also occur idiopathically [2]. A common consequence of IN is reduced visual acuity. Most patients have a null zone--a gaze angle where they have their best visual acuity. To use this gaze angle, they will turn their head in the opposite direction of the null zone (Anomalous head posture, AHP). Others may have associated strabismus.

To date, the pathogenesis of IN remains unclear. Many patients show an X-linked inheritance pattern. Two major genes, namely, FERM domain-containing 7(FRMD7) and G-protein coupled receptor 143 (GPR143), have been implicated in IN and map to regions Xp22.3-p22.2 and Xq26-q27 respectively [3]. FRMD7 contains 12 exons, encodes a 714-amino acid protein and is associated with X-linked IN [2]. Mutations associated with IN include missense mutations, null mutations, deletions and insertions, and frameshift mutations. Mutations in FRMD7 are the most common causes of Chinese familial X-linked IN [4].

In this study, we found a 7-bp deletion(c.823-829delACCCTAC) in the 9th exon of FRMD7 in a Chinese family with IN, which predicted a truncated protein. Our data expands the spectrum of FRMD7 mutations causing IN.

## Methods

### Patients

A Chinese family with idiopathic infantile nystagmus (IIN) was recruited at the Department of Ophthalmology in XinHua hospital, Shanghai Jiao Tong University. All patients received comprehensive ophthalmic examinations, and eye movements were recorded. Then,5-ml of blood samples were collected to conduct high-throughput sequencing. This study was approved by the Ethics Committee of the XinHua hospital and conformed to the tenets of the Declaration of Helsinki. Informed consent was obtained from all participants. (If the patient was a minor, the informed consent was signed by parents or guardian)

### Ophthalmic examinations

A comprehensive ophthalmic examinations was performed on each subject. We used Snellen visual acuity charts and the Titmus test to measure visual acuity and stereopsis. Visual acuity was measured monocularly and binocularly for distance both at the patient’s AHP and corrected posture with the patient’s best optical correction in place. The Hirschberg test and the prism cover test were used to assess for strabismus. We used a divider with a linear calibration (goniometer) to measure the degree of AHP when patients looked at a target from 5-m away. One arm of the goniometer was in the sagittal direction of the head. The other arm was in the sagittal direction of the body. The angle of the corner of the two arms defined the angle of the AHP. Horizontal and vertical eye-movement measurements were taken using IR reflection with a sampling frequency of 500 Hz. Calibration was performed with the opposite eye covered. The patient was required to fixate horizontally from 0 to 30° in 5-degree increments and vertically from 0 to 20° both monocularly and binocularly. The position of the null zone was determined by eye movement recordings. The duration of each examination was approximately 10 seconds. Ocular examinations also included cycloplegic refraction, slit-lamp examination, electroretinography and visual evoked potential response. From fundus photographs, we analyzed each patient’s retinal vasculature, retinal pigment and the morphology of the optic nerve head, including the peripapillary double ring sign. We also measured the cup-to-disc ratio (C/D ratio) and the disc–macula distance to disc diameter ratio (DM:DD ratio). When the DM:DD ratio was greater than 3.0, a small optic disc could be indicated.

### Mutation analysis

Mutations in the FRMD7 gene were screened by high-throughput sequencing using genomic DNA from the patient’s peripheral blood. We used a DNA extraction kit (TIANGEN, Beijing, China) according to the manufacturer’s instructions. DNA was quantified using a Nanodrop 2000 PCR-amplification of all of FRMD7’s all coding exons and intron-exon junctions was performed with the primers according to a previous report [5]. PCR products were sequenced on an ABI PRISM 3730XL DNA analyzer (Applied Biosystems, Foster,CA,USA). The sequences were aligned, and the mutations were detected using mutation surveyor software (Softgenetic,Pennsylvania,USA). The effects of the mutations on the protein coding region (synonymous, missense, nonsense, frameshift, etc.) were predicted by Exome-assistant, and the potential pathogenic effects of the mutations on protein function were estimated using Mutation Taster (http://www.mutationtaster.org), Polyphen-2 (http://genetics.bwh.harvard.edu/pph2/) and SIFT (http://sift.jcvi.org/www/SIFT_enst_submit.html). The database we used were the ExAC (http://exac.broadinstitute.org) ,the exome Variant Server (http:// evs.gs.washington.edu/EVS/), and the 1000 Genomes Project (http://www.1000genomes.org/).

## Results

### Clinical characteristics

There were 20 people in this Chinese family; 10 of them had IN (5 female and 5 male). All IN patients we examined presented nystagmus within the first six months of life, conjugate horizontal oscillation of the eyes, reduced visual acuity and stereopsis. The mean LogMAR binocular visual acuity measured at both their AHP and corrected head posture was 0.25 and 0.37, respectively. No patient had associated strabismus. Four patients had obvious AHPs and two chose to receive surgery to shift the null zone. Four had similar nystagmus wave forms(bidirectional jerk, BDJ). One patient had pure jerk nystagmus (J), jerk nystagmus with extended foveation (JEF) and bidirectional jerk nystagmus (BDJ). Another had pseudo-pendular nystagmus with foveating saccades (PPFS). None had anterior-segment pathologies or macular hypoplasia. The mean C/D ratio was 0.35± 0.14 (right eye) and 0.39± 0.10 (left eye). The mean DM/DD ration was 2.66± 0.77 (right eye) and 2.43± 0.68 (left eye). No one had obvious morphological changes of the optic nerve head. However, one patient’ s DM:DD ratio was greater than 3.0.

### Identification of the FRMD7 mutation

**Fig. 1:**
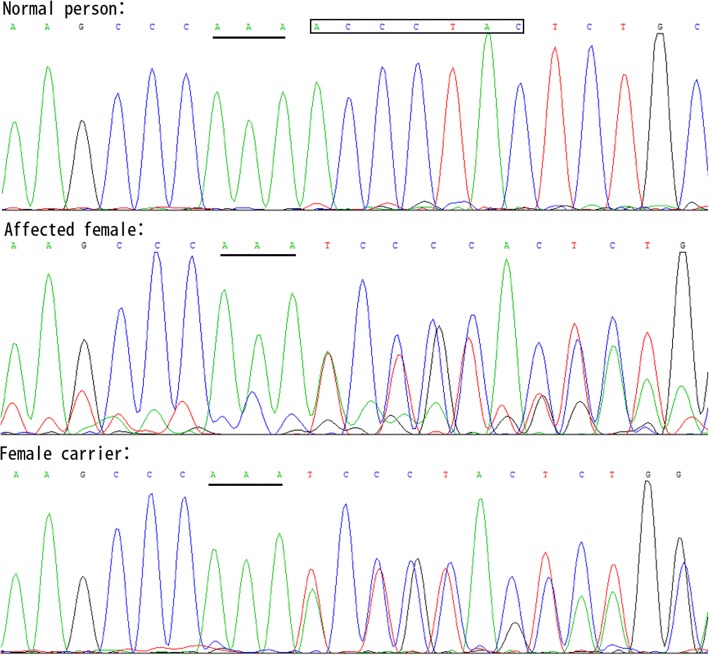
Sequencing chromatograms. Affected female and female carriers show a 7bp deletion c.823-829delACCCTAC, causing a frameshift mutation at codon 275(p.Thr275fs)

**Fig. 2:**
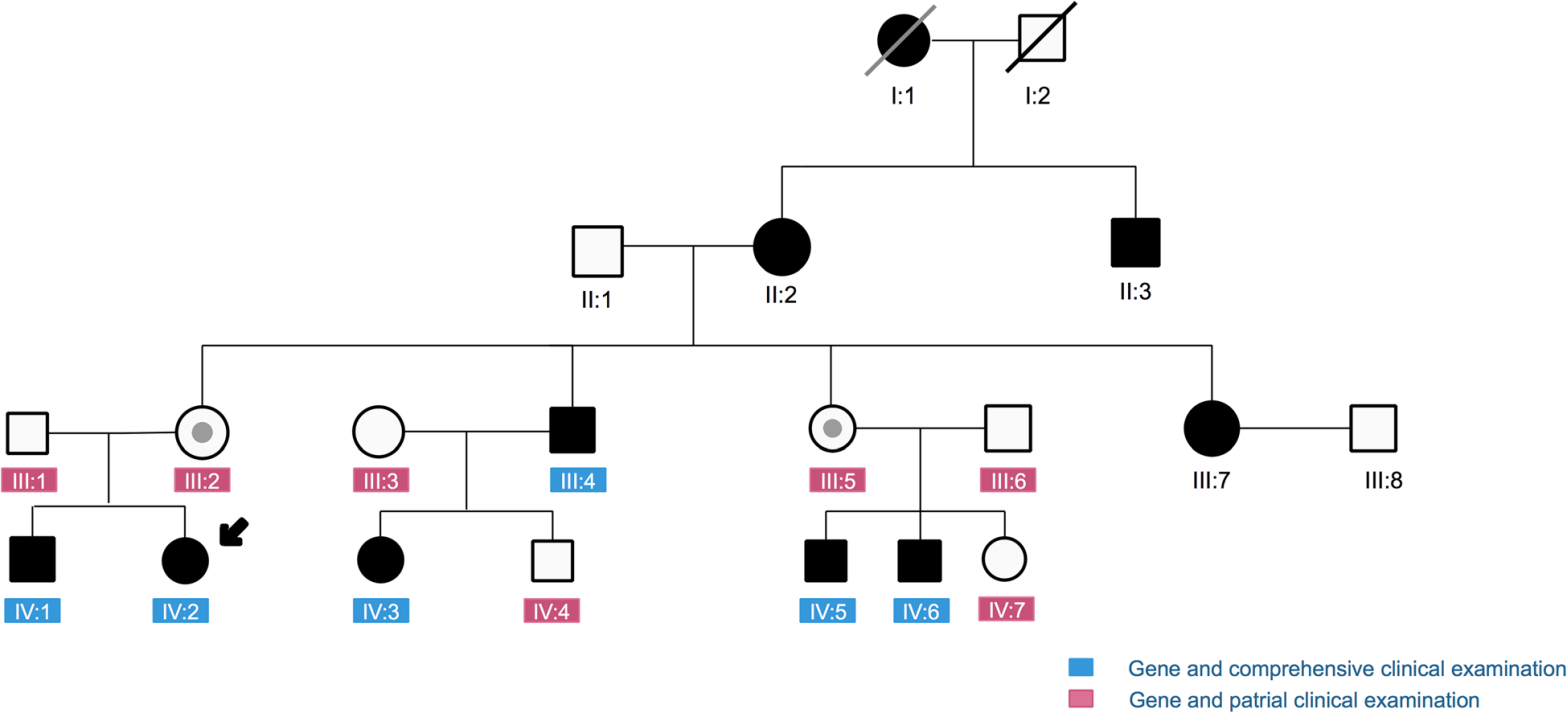
Pedigrees of this Chinese family. Filled symbols demonstrate affected patients and unfilled symbols demonstrate unaffected patients. The dotted circles show female carriers. Arrow indicates the proband. Red indicates that patients underwent a genetic test and partial clinical examination. Blue indicates that patients underwent a genetic test and complete clinical examination

Sequence analysis of this Chinese family detected a novel frameshift mutation c.823-829delACCCTAC in the 9th exon of FRMD7. This mutation results in a frameshift mutation at codon 275 (p.Thr275fs) and predicts protein truncation. This mutation was confirmed to extend to other affected family members. However, this mutation was not found in the unaffected members or in the 100 unrelated controls (Fig. 1). There was no male-to-male transmission, female carriers were heterozygous, and male carriers were hemizygous. All male carriers were affected. The genetic pattern of disease conforms to X-linked dominant inheritance (Table 1 and Fig. 2). Some female members (III-2, III-5) heterozygous for the FRMD7 mutation did not have IN. This finding suggested incomplete penetrance for this truncating FRMD7 mutation.

**Table 1:** Genotypes and phenotypes of this Chinese family

Patient ID	Sex/Age	Nystagmus	BCBVA(A/C)	Strabismus	C/Dratio (OD/OS)	DM/DD (OD/OS)	Type	AHP	Affected
III:1	M/41	N	N/0	N	NA	NA	-	N	No
III:2	F/40	N	N/0	N	NA	NA	Heterozygous	N	No
III:3	F/35	N	N/0	N	NA	NA	-	N	No
III:4	M/37	PPFS	0.2/0.4	N	0.50/0.58	3.91/3.75	Hemizygous	TR10	Yes
III:5	F/36	N	N/0	N	NA	NA	Heterozygous	N	No
III:6	M/38	N	N/0	N	NA	NA	-	N	No
IV:1	M/10	Bdj	0.4/0.4	N	0.27/0.36	2.45/2.17	Hemizygous	N	Yes
IV:2	F/6	Bdj	0.2/0.3	N	0.28/0.31	2.73/2.50	Heterozygous	TL30	Yes
IV:3	F/10	Bdj	0.2/0.3	N	0.16/0.33	2.46/2.05	Heterozygous	CU25	Yes
IV:4	M/7	N	N/0	N	NA	NA	-	N	No
IV:5	M/12	J,jef,bdj	0.3/0.4	N	0.5/0.42	1.53/1.87	Hemizygous	TR15	Yes
IV:6	M/7	Bdj	0.2/0.4	N	0.38/0.33	2.88/2.22	Hemizygous	CU20	Yes
IV:7	F/6	N	N/0	N	NA	NA	-	NA	No

*BCBVA* Binocular corrective best visual acuity. (LogMAR), *A/C* visual acuity of AHP/head straight, *C/D ration* cup-to-disc ratio, *DM/DD ration* disc–macula distance to disc diameter ratio, *TR* Face turn right, *TL* Face turn left, *CU* chin-up, *CD* chin-down, *PPFS* pseudo-pendular nystagmus with foveating saccades, *Bdj* bidirectional jerk, *J* pure jerk nystagmus, *Jef* jerk nystagmus with extended foveation, *N* normal, *NA* not available, *F* female, *M* male

## Discussion

In this study, we found a novel frameshift mutation in the FRMD7 gene. FRMD7 contains 12 exons and encodes a 714-residues polypeptide. FRMD7, which contains the FERM-N, FERM-M, FERM-C and FA structural domains in the NH2-terminus, is a member of the 4.1 superfamily [6, 7]. The FRMD7 protein is conserved near the NH2-terminus that includes the B41 and FERM-C domains. Residues 1-192 contain the B41 domain, and residues 186-279 contain the FERM-C domain [8].

FRMD7 has been shown to regulate neuronal outgrowth by influencing the dynamics of F-actin during retinoic acid-induced differentiation in mouse neuroblastoma cells [9]. The FRMD7 gene is expressed in the midbrain, spinal cord, cerebellar primordium, the ventricular layer of the forebrain, and the developing neural retina in human embryos [10]. It is known to influence the motor control of eye movement, and Betts-Henderson et al. found that FRMD7 may play an important role in multiple aspects of neuronal development [11]. It has been reported that the FRMD7 protein interacts with calcium/calmodulin-dependent serine protein kinase (CASK) and connects the actin cytoskeleton to the plasma membrane [12].The activity of the FRMD7 protein can be abolished by affecting its stability or its binding with interacting partners. Thus, FRMD7 mutation may cause nystagmus by damaging neuronal activity in the area of the brain that controls eye movement [13].

Idiopathic INS patients with FRMD7 mutation are not known to have structural abnormalities in the afferent visual pathway. Although our patients with FRMD7 mutations had diminished visual acuity, they were similar to those previously described [14, 15]. This mild visual loss may be attributed to the assumption that the FRMD7 mutations cause defects in the central nervous system, where ocular motor function is controlled. However, recent studies have shown anomalies of the optic nerve or retina, including a shallow foveal pit, increased central-macular thickness, decreased peripapillary-retinal nerve-fiber layer thickness, shallow optic-nerve cup and optic-nerve dysplasia in IIN patients with FRMD7 mutations [14, 15]. Furthermore, another study demonstrated that FRMD7 expression has been localized to starburst cells of the retina, and FRMD7 mutant mice lost asymmetric inhibitory inputs to horizontal direction-sensitivity cells, abolishing the optokinetic reflex. These results indicated that FRMD7 mutations are associated with the abnormal development of the afferent visual pathway, and the FRMD7 protein is necessary for the establishment of neuronal circuit asymmetries [16].

Nystagmus was transmitted as an apparent X-linked dominant with incomplete penetrance trait in our family with five affected females, two obligate female carriers, five affected males, and no male-male transmission. In X-linked congenital idiopathic nystagmus pedigrees, penetrance among female members has been variable, ranging from 30% to 100% [17, 18]. The mechanism of incomplete penetrance of this disease in females is currently not understood. Possible mechanisms for this variable penetrance include skewed X inactivation, genetic modifiers (such as polymorphisms in interacting proteins), regulation of other genes and other non-genetic developmental influences (such as the environment) on ocular motor development. These factors may also explain why X-linked dominant and recessive pedigrees, with nystagmus or other ocular diseases, can both show linkage to the same region [17, 19].

To date, more than 66 mutations within FRMD7 have been reported [4, 6, 8, 13, 20–25]. About half of the mutation are missense and the other are nonsense mutations, frameshift mutations caused by deletion or insertion and aberrant splicing. Most mutations (75%) are unique, and always found within a family. However, most of these mutations are concentrated in the FERM and FA domains. This demonstrates that these regions play important roles in the function of FRMD7.

In our study, we found a novel frameshift mutation c.823-829delACCCTAC located in the 9th exon of FRMD7. This mutation results in a frameshift mutation at codon 275 (p.Thr275fs) and predicts protein truncation in the FERM-C domain. Another mutation was identified at this location, but that mutation was a missense mutation ( c.824A>C) [23].The FERM domain has a three-lobed “clover-leaf” structure (FERM-N, FERM-M, FERM-C) with the three domains in close proximity to each other indicating that they do not function independently. Furthermore, the FERM-C domain is highly conserved [8]. The truncation predicted by the frameshift mutation at codon 275 is likely to destabilize the protein.

This study has potential limitations. We did not screen for foveal hypoplasia and optic nerve changes quantitatively using OCT imaging. OCT imaging can provide a more detailed analysis of afferent visual systems than fundus photography. Furthermore, we did not study protein and cell level. Although the exact function of FRMD7 and the underlying molecular causes of IN are not fully understood, the spectrum of FRMD7 mutations can provide some insights.

## Conclusion

In summary, our study expanded the spectrum of FRMD7 mutations in associated with X-linked IN and confirmed that mutations in FRMD7 are the underlying molecular cause for X-linked IN.

## Abbreviations

IN: infantile nystagmus
FRMD7: FERM domain-containing 7 gene
